# Diagnostic tools of heart failure with preserved ejection fraction: Comparison of left atrial strain to the HFA-PEFF score

**DOI:** 10.21542/gcsp.2025.21

**Published:** 2025-05-15

**Authors:** Antit Saoussen, Fekih Ridha, Bahri Khalil Khalil, Ferchichi Olfa, Dridi Kalthoum, Zakhama Lilia

**Affiliations:** 1 University of Tunis El Manar, Faculty of Medicine of Tunis, Department of Cardiology, Interior security forces hospital, La Marsa, Tunisia; 2 Hematology and biology department, Internal Security Forces Hospital of Marsa, Tunisia

## Abstract

**Introduction:** Diagnosing heart failure with preserved ejection fraction (HFpEF) remains challenging. Several diagnostic criteria have been proposed, and current guidelines recommend using the HFA-PEFF score in the diagnostic algorithm for HFpEF. We sought to evaluate the clinical utility of left atrial strain (LAS) in the diagnosis of HFpEF as assessed by the HFA-PEFF score.

**Methods:** This was a prospective, mono-centric, cross-sectional study conducted from October 2021 to June 2022 in the Cardiology Department of the Internal Security Forces Hospital of Marsa, Tunisia. Patients were classified into two groups (A and B) based on the HFpEF diagnosis assessed by the HFA-PEFF scoring system: Group A with a score of ≥5 and Group B with a score of <5.

**Results:** A total of 110 patients were eligible for the study. The mean age was 61 ± 11 years. A female predominance was noted, with 57% of the patients being female. Hypertension and diabetes were the most common cardiovascular risk factors, found in 81.8% (*n* = 90) and 54.5% (*n* = 60) of patients, respectively. The median HFA-PEFF score was 4 [2-6]. Forty-six patients (41.6%) were given a clinical diagnosis of HFpEF. LAS analysis showed that PALS (*p* < 0.001) and PACS (*p* < 0.001) were significantly lower in Group A compared with Group B. PALS was strongly correlated with the HFA-PEFF score (r = −0.693, *p* < 0.001). PALS (AUC = 0.889; *p* < 0.001) was significantly the best predictor of HFpEF diagnosis. After multivariate analysis, PALS (HR = 0.782; 95% CI [0.629–0.973]; *p* = 0.027) was an independent predictor of HFpEF diagnosis, with a cut-off value of 24% (sensitivity of 86% and specificity of 89.5%).

**Conclusion:** PALS is a simple and sensitive ultrasound parameter that can be used for the diagnosis of HFpEF.

## Introduction

Heart failure with preserved ejection fraction (HFpEF) accounts for more than half of all heart failure hospital admissions^1^. Providing effective management is a major unmet clinical need that depends on a clear diagnosis.

Diagnosing HFpEF remains challenging. Several diagnostic criteria have been proposed by various societies and clinical trials, and these criteria vary widely in their sensitivities and specificities for diagnosing HFpEF. More recently, a score-based algorithm called HFA-PEFF has been proposed to aid in diagnosis^2^. This score includes pre-test evaluation, score calculation based on echocardiography and natriuretic peptide levels, functional testing (such as exercise echocardiography or hemodynamic catheterization), and a final etiology assessment.

The left atrium plays a crucial role in modulating left ventricular (LV) filling by acting as a reservoir for venous return during systole and a pump to boost LV ejection during late diastole through atrial contraction. Left atrial dysfunction is commonly seen in patients with heart failure^3^. Increasing evidence suggests that left atrial dysfunction is an important contributor to elevated LV filling pressure (LVFP), a key feature of HFpEF.

The inverse correlation of left atrial strain (LAS) with resting LVFP has been well-validated in select populations^4–6^. Current guidelines recommend using invasive or non-invasive stress testing in the diagnostic algorithm for HFpEF^2,7^. Two recent studies demonstrated the correlation of LA strain with exercise pulmonary artery wedge pressure [8] and the diagnostic value of LA strain for invasively verified HFpEF in patients referred for right heart catheterization^8^.

We sought to evaluate the clinical utility of LAS in the diagnosis of HFpEF as assessed by the HFA-PEFF score.

## Methods

### Patients and study design

This was a prospective, monocentric, cross-sectional study conducted from October 2021 to March 2022 in the Cardiology Department of the Internal Security Forces Hospital of Marsa, Tunisia. The study was approved by the hospital’s ethics committee, and informed verbal consent was obtained from all patients before participation.

We included consecutive patients presenting with exertional symptoms who were assessed at our cardiology outpatient clinic during the mentioned period and had no known structural heart diseases, such as significant valvular heart disease, prior valve replacement or repair, left ventricular ejection fraction (LVEF) <50%, infiltrative or hypertrophic cardiomyopathy, constrictive pericarditis, congenital heart disease, or idiopathic pulmonary hypertension.

**Figure 1.**
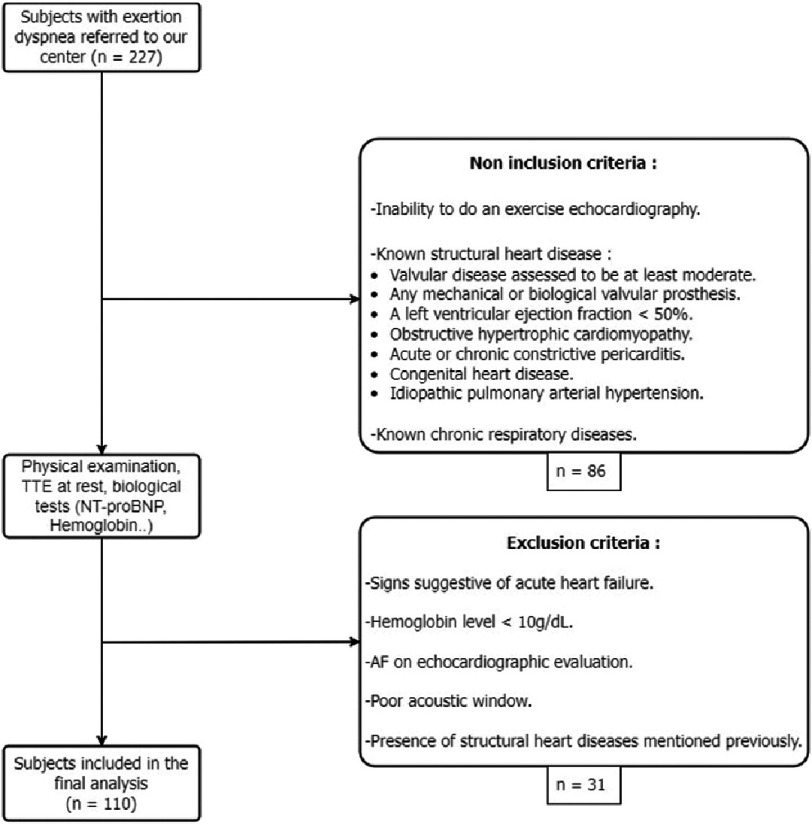
The study flow chart. TTE, Transthoracic Echocardiography; AF, Atrial Fibrillation.

Patients were excluded if they had acute heart failure, were in a rhythm other than sinus at the time of the ultrasound examination, had severe anemia, or were unable to perform exercise stress echocardiography (ESE) if indicated. Patients in whom one of the above-mentioned heart diseases was discovered on resting transthoracic echocardiography (TTE) were also excluded from our study (Figure 1).

Patient demographic and anthropometric data, cardiovascular risk factors, and comorbidities were collected. Symptoms leading to the diagnosis of heart failure, such as dyspnea, asthenia, and reduced exercise tolerance, were noted. Systolic blood pressure (SBP), diastolic blood pressure (DBP), and heart rate (HR) at rest and during exercise were measured.

All patients underwent a biological examination, which included measurements of hemoglobin (Hb), N-terminal pro-brain natriuretic peptide (NT-Pro BNP), and creatinine levels, with creatinine clearance calculated using the Modification of Diet in Renal Disease (MDRD) formula^9^.

After clinical and biological examinations conducted at our cardiology outpatient clinic, an ultrasound assessment was immediately performed at rest, followed by the initial calculation of the HFA-PEFF score at our cardiology department. Patients with low or intermediate scores underwent ESE with calculation of the final HFA-PEFF score. Patients were then classified into two groups (A and B) based on the HFpEF diagnosis assessed by the HFA-PEFF scoring system: Group A with a score of ≥5 and Group B with a score of <5.

### Echocardiographic analysis

All patients underwent an ultrasound examination at rest using a Philips EPIQ 7C echocardiography machine with simultaneous and continuous electrocardiographic tracing. All measurements were performed in accordance with the recommendations of the American Society of Echocardiography and the European Association of Cardiovascular Imaging (ASE/EACVI)^10^.

The following ultrasound parameters were collected at resting transthoracic echocardiography: left ventricular ejection fraction (LVEF), left ventricular end-diastolic diameter (LVED), left ventricular mass index (LVMI), left ventricular end-diastolic volume index (LVEDV), maximum velocities of the E wave (E) and A wave (A), E/A ratio, septal e’ velocity, lateral e’ velocity, tricuspid regurgitation maximum velocity (TR maxV), left atrial volume index (LAVI), average E/e’ ratio, and left ventricular global longitudinal strain (GLS).

**Figure 2.**
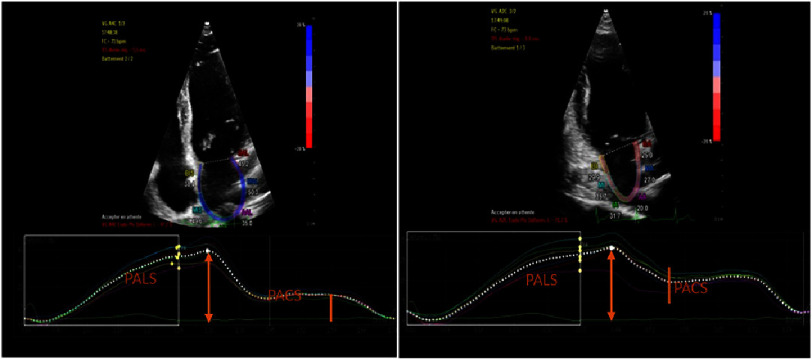
Left atrial strain measurement in four and two-chambers views. PALS, peak atrial longitudinal strain; PACS, peak atrial contraction strain.

Left atrial strain (LAS) was measured as follows: Two loops of the left atrium were obtained in apical four-chamber and two-chamber views. This was done after adjusting the focus to the left atrium and reducing the size of the sector to emphasize the distinction between the myocardium and extracardiac structures. The measurement was performed during a brief apnea with stable electrocardiogram recording and a frame rate between 60 and 80 frames per second. Atrial strain curves were generated after semi-automatic tracing of the endocardial limits (see Figure 2).

A single qualified operator in cardiac ultrasound was assigned for the analysis of left atrial strain (after loop acquisition). Two measurements were performed, and the average left atrial strain value was calculated.

Peak atrial longitudinal strain (PALS) is defined as the first peak of the positive atrial longitudinal strain at the beginning of the QRS complex. Peak atrial contraction strain (PACS) is defined as the second positive peak, which is lower than the first and corresponds to the period before atrial contraction(after the onset of the P wave on the electrocardiogram). The PALS and PACS values analyzed were averaged over apical four-chamber and two-chamber views. The normal values for PALS and PACS were 39.4% (27.6 to 59.8%) and 17.4% (14 to 25%), respectively^11^.

Exercise stress echocardiography (ESE) was performed according to the recommendations of the European Society of Cardiology^2^ using the following protocol:

• On a semi-supine bicycle, the patient pedaled at a speed of 60 rpm, starting with a low workload of 15W and gradually increasing by 5W every minute until reaching a submaximal target heart rate of 100–110/min (before the fusion of the E and A waves) OR until the patient developed limiting symptoms.

In both cases, the effort was considered maximal, and at this time, the E wave, A wave, lateral e’ velocity, septal e’ velocity, TR maxV, and the average E/e’ ratio were measured.

### Calculation of the diagnostic Score of HFpEF

Details of the score calculation in the present study are shown in Table 1. In brief, the pre-exercise score is the sum of points from various components calculated using resting echocardiographic parameters and NT-pro BNP levels, according to major (2 points) and minor (1 point) criteria. Patients were classified into three groups based on their scores: low-score (≤1 point), intermediate-score (2–4 points), and high-score (≥5 points).

**Table 1:** HFpEF score calculation according to consensus recommendation from the Heart Failure Association (HFA) of the European Society of Cardiology^2^.

**Criteria**	**Functional**	**Morphological**	**Biomarker**
Major	Septal e’ < 7 cm/s or lateral e’ < 10 cm/s Or Average E/e’ ≥ 15 Or TR velocity > 2.8 m/s (PASP > 35 mmHg	LAVI > 34 ml/m^2^ Or LVMI ≥ 149/122 g/m^2^ (m/w) and RWT > 042	NT-proBNP > 220 pg/ml Or BNP > 80 pg /ml
Minor	Average E/e’ 9–14 Or GLS < 16%	LAVI 29–34 ml/m^2^ or LVMI > 115/95 g/m^2^ (m/w) or RWT > 042 Or LV wall thickness ≥ 12 mm	NT-proBNP 125–220 pg/ml Or BNP 35–80 pg /ml
**Major criteria: 2 points**		**Score: ≥ 5 points: HFpEF**	
**Minor criteria: 1 point**		**Score: 2–4 points, consider diastolic stress test or invasive hemodynamic measurements**	

**Notes.**

GLS global longitudinal strain BNP Brain natriuretic peptide HFpEF Heart failure with preserved ejection fraction LAVI left atrial volume index LV left ventricular LVMI left ventricular mass index PASP Pulmonary artery systolic pressure RWT relative wall thickness TR tricuspid regurgitation

The algorithm recommends noninvasive or invasive exercise testing for patients with intermediate scores. Exercise testing can add additional points to the score: 2 points for a mitral inflow average E/e’ ratio ≥ 15 and 1 additional point for a tricuspid regurgitation maximum velocity (TR maxV) > 3.4 m/s. A final score of ≥5, whether at rest or after exercise, indicates a high likelihood of HFpEF and is considered equivalent to a diagnosis of HFpEF.

### Statistical analysis

Data were recorded and analyzed using IBM SPSS Statistics 23 software. Baseline characteristics were summarized as mean ±  standard deviation (SD) or median ± 25th–75th percentiles for continuous data, and counts with percentages for categorical data. The Kolmogorov–Smirnov test was used to assess the normality of continuous variable distributions.

Comparisons of means between independent groups were performed using the Student’s *t*-test. Comparisons of percentages between independent groups were conducted using the Pearson chi-square test. The relationships between two quantitative variables were assessed using Pearson’s correlation coefficient (r). Correlations were categorized as very weak (r = 0−0.19), weak (*r* = 0.2−0.39), moderate (*r*=0.4−0.59), strong (*r* = 0.6−0.79), and very strong (*r* = 0.8−1.0). The threshold values of the studied parameters were determined by analyzing their receiver operating characteristic (ROC) curves and comparing the areas under the curve (AUC) using the Delong method.

Binary logistic regression was performed for multivariate analysis using the significant parameters from the univariate analysis. A significance level of 0.05 was set for all statistical tests.

## Results

### General characteristics

A total of 110 patients were eligible for the study. Patient characteristics are summarized in Table 2. The mean age was 61 ±11 years. There was a female predominance with 63 female patients (57%) and 47 male patients (43%). Hypertension and diabetes were the most common cardiovascular risk factors, found in 81.8% (*n* = 90) and 54.5% (*n* = 60) of patients, respectively. Twenty patients (18%) had coronary artery disease, and 86.4% (*n* = 95) were either overweight or obese. The most frequent complaints were exertional dyspnea (88%, *n* = 97) and poor exercise capacity (20%, *n* = 22).

**Table 2:** Comparison of clinical, biological, and ultrasonographic characteristics between the two groups.

	General population (110 = 100%)	Group A (46 = 41, 8%)	Group B (64 = 58, 2%)	*P* value
Sex Male/Female (%)	47 (42.7)/63 (57,3)	13 (28.3)/33 (71,7)	34 (53)/30 (47)	**0.009**
Age (years)	60 ± 11	69 ± 9	56 ± 10	**<0.001**
Hypertension (n %)	90 (81.8)	42 (91.3)	48 (75)	**0.029**
Smoking (n %)	21 (19.1)	5 (11)	16 (25)	0.063
Dyslipidemia (n %)	47 (42.7)	17 (37)	30 (47)	0.343
Diabetes (n %)	60 (54,5)	30 (65)	30 (46)	**0.045**
CKD (n %)	9 (8.2)	9 (16.6)	0	**<0.001**
Overweight/obesity (n %)	95 (86.4)	38 (82.6)	57 (89)	0.331
Coronary artery disease (n %)	20 (18.2)	11 (24)	9 (14)	0.220
SBP (mmHg)	140 [130–145]	145 [135–150]	135 [120–140]	**0.001**
DBP (mmHg)	80 [75–90]	83 [80–90]	80 [75–84]	0.348
Heart ate (b/m)	79 [70–84]	80 [70–86]	77 [71–84]	0.574
Creatinine level (*μ*mol/l)	70 [57–89]	85 [63–100]	63 [53–73]	**<0.001**
Creatinine clearance (ml/min/1.73m^2^ )	92 ± 32	74 ± 29	104 ± 29	**<0.001**
Hemoglobin level (d/dl)	12.7 ± 1.4	12.1 ± 1.5	13.2 ± 1.2	**<0.001**
NT Pro-BNP (pg/ml)	86.5 [52–247]	241 [126–422]	52 [28–78]	**<0.001**
LVEF (%)	64 ± 6	63 ± 7	65 ± 6	0.172
LVED (mm)	49 ± 5	50 ± 5	49 ± 6	0.134
LVEDV (ml/kg/m^2^ )	42 [36–51]	40 [35–46]	43 [35–47]	0.761
LVMI (g/m^2^ )	88 ± 22	98 ± 24	83 ± 19	0.172
LAVI (ml/m^2^ )	37 ± 11	44 ± 12	31 ± 7.3	**<0.001**
Rest E/A ratio	1.06 ± 0.42	1.2 ± 0.54	0.95 ± 0.28	**0.001**
Rest average e’ (cm/s)	8.6 ± 2.4	7.2 ± 1.7	10 ± 2.1	**<0.001**
Average E/e’ ratio at rest	10,3 ± 3.9	13 ± 3.1	7,8 ± 1.7	**<0.001**
TR velocity at rest (cm/s)	2.5 ± 0.4	2.8 ± 0.5	2.2 ± 0.3	**<0.001**
GLS (%)	-22 [-24_-20]	-22 [-24_-20]	−22.8 [-24_-22]	0.066
PALS %	27 [21–32]	21 [17–23]	32 [29–33]	**<0.001**
PACS (%)	13.7 ± 4	10.6 ± 4	16 ± 4	**<0.001**
Average E/e’ ratio at exercise	9.3 ± 3	17 ± 1	8.4 ± 2.1	**<0.001**
TR velocity at exercise (cm/s)	2.8 ± 0.5	3.2 ± 0.3	2.6 ± 0.5	**0.036**

**Notes.** Data are presented as mean ± SD, median (interquartile range), or count and percentage of patients. Bold values indicate *p* < 0.05.

CKD chronic kidney disease SBP systolic blood pressure DBP diastolic blood pressure LVEF left ventricular ejection fraction LVED left ventricular end-diastolic diameter LVEDV left ventricular end-diastolic volume LVMI left ventricular mass index LAVI left atrial volume index TR tricuspid regurgitation GLS global longitudinal strain PALS peak atrial longitudinal strain PACS peak atrial contraction strain

The mean hemoglobin value was 12.7 ± 1.4 g/dL, while the median values for NT-Pro BNP and creatinine were 96 [52–247] pg/mL and 93 [66–114] µmol/L, respectively. Nineteen patients (17%) had creatinine clearance <60 mL/min.

The mean left ventricular ejection fraction (LVEF) was 64 ±6%. The median global longitudinal strain (GLS) was -22% [−24% to −20%]. The median peak atrial longitudinal strain (PALS) value was 27% [21–32%], and the mean peak atrial contraction strain (PACS) value was 13.7% ± 4%.

The median value of the final HFA-PEFF score was 4 [2–6]. Among the 110 patients, 37 (33.6%) had a high score, 55 (50%) had an intermediate score, and 18 (16.4%) had a low score before undergoing exercise testing. In those with intermediate scores according to resting data, 9 patients (16%) developed elevations in left ventricular filling pressure (LVFP) during exercise. Consequently, 46 patients (41.8%) were given a clinical diagnosis of HFpEF based on the ESC diagnostic algorithm.

**Figure 3.**
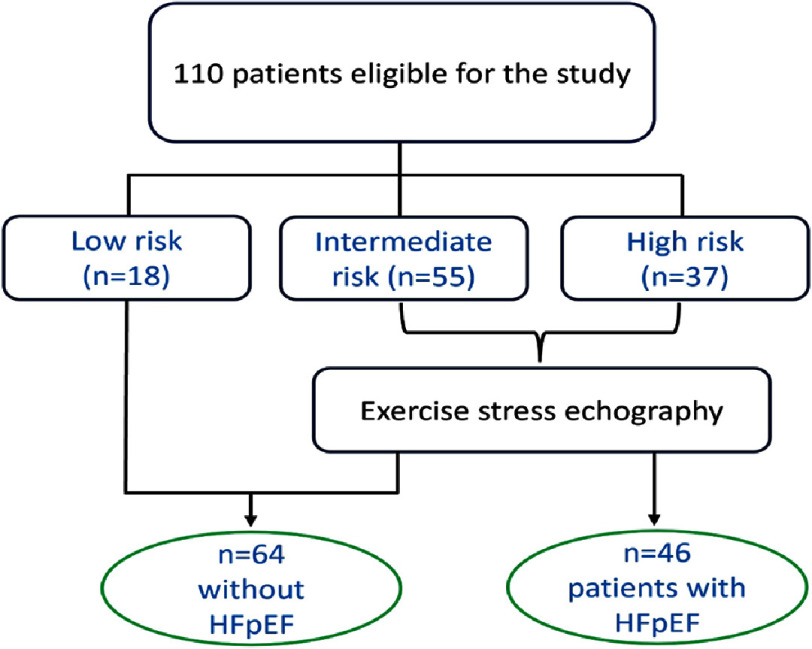
Protocol of patient’s repartition based on the HFA-PEFF score. HFpEF, heart failure with preserved ejection fraction.

Figure 3 illustrates the final distribution of patients based on the HFA-PEFF score.

### Correlation between left atrial strain and the HFA-PEFF score

Based on these findings, patients were categorized into two final groups according to the HFA-PEFF score: Group A (46 patients, 41.8%) and Group B (64 patients, 58.2%). A comparison of clinical, biological, and ultrasonographic characteristics between these groups is detailed in Table 2.

Patients in Group A were significantly older (*p* < 0.001), had a higher proportion of females (*p* = 0.009), and showed a significantly higher prevalence of hypertension (*p* = 0.029), diabetes (*p* = 0.045), and chronic kidney disease (*p* < 0.001). Systolic blood pressure (SBP) was also significantly higher in this group (*p* = 0.001).

Group A patients had elevated creatinine levels (*p* < 0.001), reduced creatinine clearance (*p* < 0.001), and lower hemoglobin levels (*p* = 0.031). NT-Pro BNP levels were significantly higher in Group A (*p* < 0.001).

Ultrasound parameters related to diastolic function were significantly associated with a high HFA-PEFF score, including the E/A ratio (*p* = 0.001), average E/e’ ratio at rest (*p* < 0.001), TR maxV at rest (*p* < 0.001), average e’ velocity at rest (*p* < 0.001), left atrial volume index (LAVI) (*p* < 0.001), average E/e’ ratio during stress (*p* < 0.001), and TR maxV during stress (*p* = 0.036).

Left atrial strain (LAS) analysis revealed that peak atrial longitudinal strain (PALS) (*p* < 0.001) and peak atrial contraction strain (PACS) (*p* < 0.001) were significantly lower in Group A compared to Group B.

**Figure 4.**
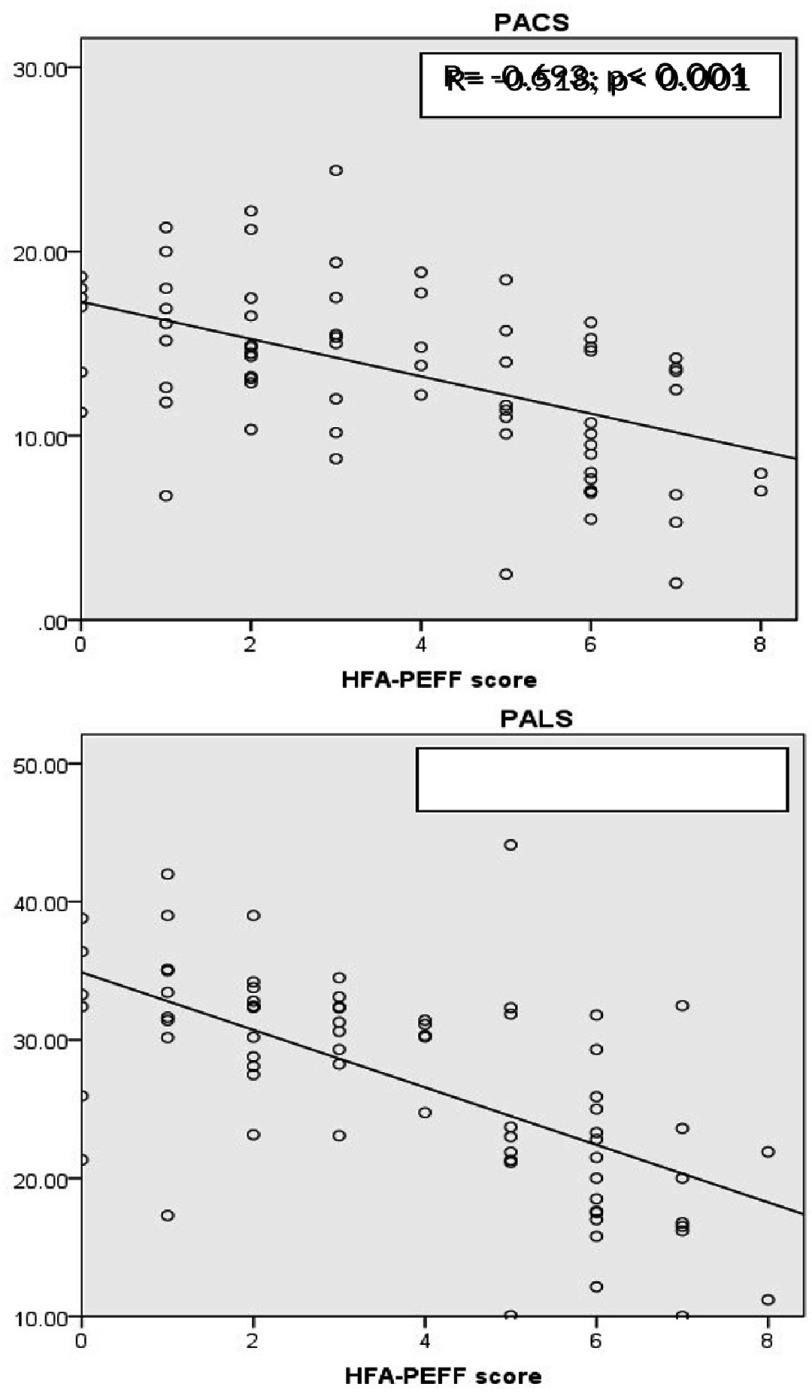
Bivariate analysis. PALS, peak atrial longitudinal strain; PACS, peak atrial contraction strain.

To explore the relationship between LAS and the HFA-PEFF score further, a bivariate analysis was performed, as shown in Figure 4. LAS was significantly and negatively correlated with the HFA-PEFF score (*p* < 0.001). The correlation was strong for PALS (r =−0.693) and moderate for PACS (r = −0.518).

**Figure 5.**
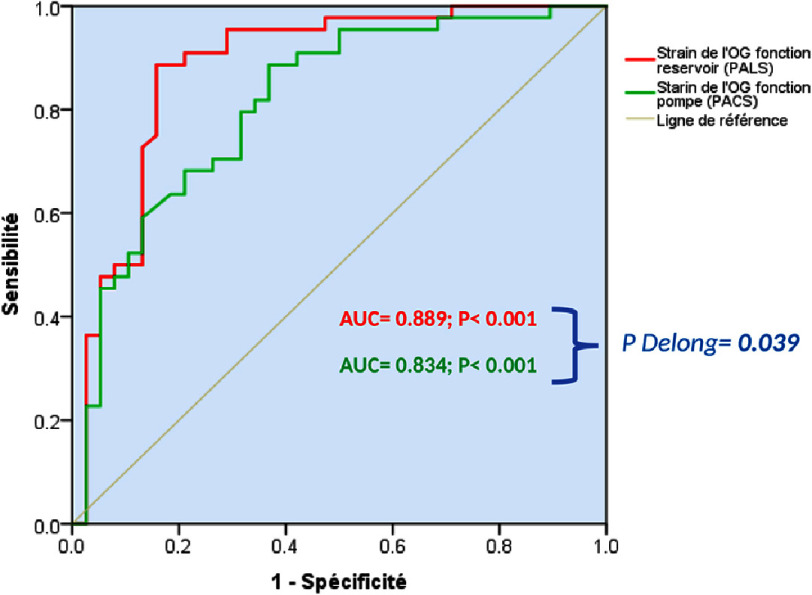
ROC curve analysis to assess the performance of left atrial strain components in the prediction of HFpEF diagnosis. AUC, area under the curve; PALS, peak atrial longitudinal strain; PACS, peak atrial contraction strain, Ligne de référence, reference line.

ROC curve analysis assessed the performance of LAS components in predicting HFpEF diagnosis (Group A) (Figure 5). Both LAS components performed well in detecting high LVFP. PALS (AU*C* = 0.889; *p* < 0.001) was the best predictor for Group A, and its superiority was significant when compared with the Delong method (*p* = 0.039).

ROC curve analyses identified cut-off values for LAS components predicting HFpEF diagnosis. A PALS score of <24% (HR = 34; 95% CI [20–117]; *p* < 0.001; sensitivity 86%, specificity 89.5%, positive predictive value 84%, and negative predictive value 90%) increased the likelihood of being in Group A. For PACS, a cut-off value of <13% (HR = 8.5; 95% CI [3–20]; *p* = 0.001; sensitivity 68%, specificity 77%, positive predictive value 72%, and negative predictive value 74%) increased the likelihood of being in Group A.

The binary logistic regression analysis included all parameters with univariate *p*-values <0.05, except for those used in the HFA-PEFF score calculation. The analysis found that only PALS (HR = 0.782; 95% CI [0.629–0.973]; *p* = 0.027) was an independent predictor of HFpEF diagnosis, as shown in Table 3. According to this model, there was a 22% higher odds of having HFpEF per 1% decrease in PALS. Female sex, age, diabetes, hypertension, CKD, SBP, creatinine levels, creatinine clearance, E/A ratio, and PACS were dependent variables.

**Table 3:** Multivariate analysis.

**Variables**	**HR**	**95% CI**	**P value**
**Female sex**	11	0.51–253	0.125
**Age**	1.07	0.96–1.11	0.178
**Diabetes**	1.39	0.25–7.66	0.699
**Hypertension**	0.31	0.03–3.11	0.325
**CKD**	0.001	0–0.01	0.999
**SBP**	1.09	1.00–1.28	0.50
**Creatinine levels**	1.04	0.93–1.17	0.470
**Creatinine clearance**	0.98	0.91–1.06	0.676
**E/A ratio**	26	0.57–1198	0.093
**PALS**	**0.78**	**0.629–0.97**	**0.027**
**PACS**	1.13	0.82–1.55	0.432

**Notes.**

CKD chronic kidney disease SBP systolic blood pressure PALS peakatrial longitudinal strain PACS peak atrial contraction strain UB upper bound LB lower bound CI confidence interval HR hazard ratio

## Discussion

The major findings of the present study were:

• LAS components are moderately to strongly correlated with the HFA-PEFF score.
• The cut-off values to predict HFpEF were 24% for PALS and 13% for PACS.
• Only PALS was an independent predictor of HFpEF diagnosis.

The diagnosis of HFpEF remains challenging due to its complexity and heterogeneity. Hemodynamic evaluation of left ventricular pressures is the gold standard for confirmation, with parameters such as pulmonary capillary wedge pressure (PCWP), left ventricular end-diastolic pressure (LVEDP), pulmonary artery pressure (PAP), stroke volume (SV), and cardiac output (CO) assessed both at rest and during exercise^12,13^. However, these invasive tests have drawbacks, including potential complications^14^, high costs, and limited availability, confining their use to research settings and high-suspicion cases where therapeutic intervention is consequential^12,15^. Non-invasive tools, particularly echocardiography, are central to current practice. The 2016 diagnostic algorithm relies on signs and symptoms of heart failure, a preserved ejection fraction(LVEF ≥ 50%), and objective evidence of left ventricular diastolic dysfunction. Key echocardiographic parameters include septal and lateral e’ waves, E/e’ ratio, left atrial volume index(LAVi), and tricuspid regurgitation (TR) peak velocity. While these parameters lack sensitivity and specificity as standalone markers, their combined assessment improves diagnostic performance^16,17^. The Heart Failure Association (HFA) of the European Society of Cardiology (ESC) introduced the HFA-PEFF score in 2019, incorporating biological and echocardiographic parameters at rest and during exertion^2^. While high scores have excellent specificity, the intermediate probability group remains problematic, requiring stress testing, which is often unavailable. Additionally, NT-proBNP, a key score component, is influenced by obesity, renal impairment, and atrial fibrillation, limiting its diagnostic reliability.

Recent studies highlight LAS as a promising marker of diastolic dysfunction, consisting of three distinct phases during each cardiac cycle:

• Reservoir Phase (PALS): The left atrium acts as a reservoir during ventricular systole.
• Conduit Phase: The left atrium serves as a conduit for blood flow in early diastole.
• Booster Pump Phase (PACS): The left atrium contracts in late diastole.

PALS contributes to left ventricular filling by storing energy during systole, while PACS reflects left atrial contractility and left ventricular end-diastolic pressure ^18–20^.

Studies have demonstrated that decreased LAS correlates with worsening diastolic dysfunction and elevated LVFP, outperforming traditional markers like E/e’ ratio or LAVi^21–24^.

Singh et al. identified an optimal PALS cut-off of 24% for differentiating mild from severe diastolic dysfunction^21^. Other studies proposed varying cut-offs: Brecht et al. reported <36% (AUC 0.82)^22^, Aung et al. suggested <17.5% (AUC 0.89, sensitivity 89%, specificity 55.3%)^25^, and Morris et al. associated <23% with diastolic dysfunction and HF hospitalization risk^24^. Wakami et al. and Kurt et al. correlated LAS with invasively measured LVEDP, identifying thresholds of <30% and <31%, respectively ^26,27^. Lundberg et al. found <21% to be predictive of elevated LVEDP (AUC 0.90)^28^.

Variability in LAS cut-offs stems from differences in HFpEF definitions, sample characteristics (e.g., CAD prevalence, age), and measurement techniques. Many studies assessed patients referred for catheterization without specific HFpEF focus, often relying on resting evaluations, which may not capture early-stage disease^27,29,30^. Early-stage HFpEF presents with exertional dyspnea, normal NT-proBNP, and normal resting LVFP that rise during exercise, emphasizing the need for diastolic stress testing^31–35^.

Exercise testing enhances diagnostic accuracy, revealing latent diastolic dysfunction. Eisman et al. showed that resting LVFP can be falsely normal in 40% of HFpEF patients, necessitating exertional evaluation^36^. Non-invasive diastolic stress testing is crucial, as LVFP assessment during exercise improves diagnostic algorithms^2,32,37^. Ye et al. linked reduced PALS with lower exercise tolerance and increased LVFP upon exertion^38^. Telles et al. found that PALS and PACS correlated with invasive pulmonary capillary pressures during exercise, with PALS ≤ 33% predicting HFpEF (sensitivity 88%, specificity 77%)^35^.

The correlation between LAS and HFA-PEFF score components has been explored. Guan et al. observed significant correlations between PALS, PACS, and E/e’ ratio in HFpEF patients^39^. Abid et al. found negative correlations between PALS and both E/e’ ratio and LAVi^40^, while Frydas et al. linked PALS with E/A ratio, LAVI, E/e’ ratio, and TR maxV^41^. Additionally, LAS correlates more strongly with invasive LVFP than LAVI and pulmonary capillary pressures compared to the E/A ratio^6,42^.

Kurt et al. reported a negative correlation between PALS and NT-proBNP (r = −0.42, *p* = 0.001)^24^, a finding echoed by Topal et al. in acute coronary syndrome patients^43^. Ye et al. demonstrated that lower PALS was associated with elevated LVFP during exercise, independent of resting diastolic dysfunction^38^. Faxen et al. found that PALS negatively correlated with the HFA-PEFF score (r = −0.35, *p* < 0.001)^44^, while Katbeh et al. identified PALS as an independent predictor of high HFpEF probability (AUC 0.78, HR = 1.22)^45^.

Current ASE/EACVI guidelines recommend exercise echocardiography when resting assessment is inconclusive^7,46,47^. ESC guidelines emphasize stress testing in intermediate-risk patients^2^. However, access to stress testing is limited, and about 20% of patients requiring exercise echocardiography are either unable to exercise or have non-measurable E/e’ ratios^48,49^. Our study suggests that a PALS <24% (sensitivity 86%, specificity 89.5%) could serve as a surrogate for exercise testing, providing a reliable resting parameter reflective of LVFP under exertion.

These findings support the role of LAS, particularly PALS, as a non-invasive marker for HFpEF. Its strong correlation with hemodynamic parameters and the HFA-PEFF score suggests that it could aid in clinical decision-making when stress testing is unavailable. Further research should focus on standardizing LAS measurement techniques and integrating stress LAS assessment into routine practice.

### Perspectives and proposed algorithm

LAS reservoir function has been shown to outperform other echocardiographic parameters in the assessment of LVFP. It is closely correlated with exercise capacity and may serve as a potential predictor of diastolic function changes during exertion. As such, its evaluation could influence the final diagnostic decision, particularly when considering the results of exercise echocardiography.

**Figure 6.**
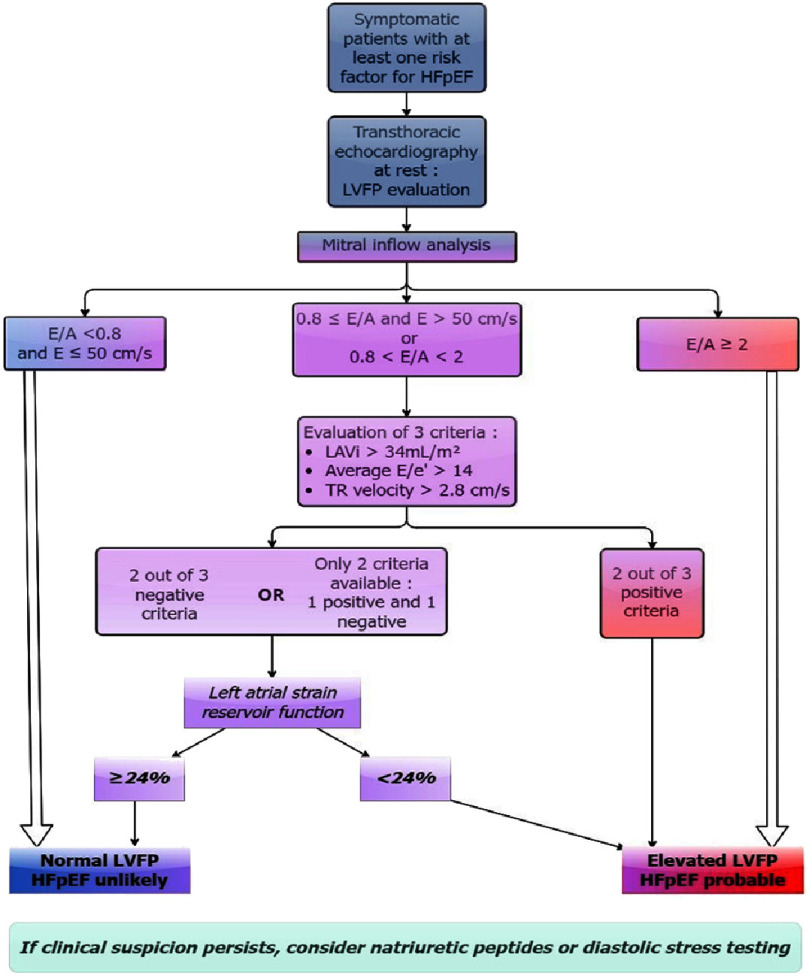
Final proposed algorithm for the diagnosis of HFpEF after incorporation of LAS reservoir function.

We propose incorporating this parameter into the 2016 diagnostic algorithm to enhance its diagnostic accuracy and performance while reducing the need for exercise testing or invasive procedures. The updated algorithm is illustrated in Figure 6.

The study of left atrial deformation thus plays a crucial role in the diagnostic work-up of patients experiencing exertional dyspnea. Once HFpEF (heart failure with preserved ejection fraction) is diagnosed, the initial therapeutic strategy focuses on diuretic therapy and optimal management of cardiovascular risk factors. Recently, the introduction of SGLT2 inhibitors has further refined this approach. Treatment has significantly improved both morbidity and mortality in HFpEF patients, underscoring the importance of early and accurate diagnosis.

It remains uncertain whether patients with early-stage HFpEF will benefit from this treatment approach, and further research is needed to clarify this. In this context, diagnostic tools like LAS will be crucial for the accurate identification and study of this subtype of heart failure.

## Limitations

We note that our study has several limitations:

***Monocentric and cross-sectional design:*** Being monocentric and cross-sectional, the study may introduce selection bias. However, we included consecutive patients to mitigate this bias.

***Lack of invasive hemodynamic validation:*** We used echocardiographic variables to define high left ventricular filling pressures (LVFP) without hemodynamic validation, which limits the diagnostic accuracy. Invasive testing, such as right heart catheterization, would provide more precise hemodynamic data.

***Interobserver and intra-observer variability:*** Although a single operator with expertise in echocardiography performed the left atrial strain (LAS) analysis, variability could still occur between measurements. Two measurements were taken, and the mean value was calculated to minimize this variability, but some degree of error remains possible.

***Small sample size:*** The relatively small sample size compared to larger-scale studies may limit the statistical power and accuracy of estimating the association between LAS and LVFP. This also affects the generalizability of our findings.

***Limited follow-up:*** As a cross-sectional study, we lack longitudinal data to assess how LAS and other echocardiographic parameters may change over time, which could provide more insight into their predictive value for HFpEF progression.

Further longitudinal studies with larger populations are needed to confirm and expand upon these results.

## Conclusion

Our findings align with recent studies highlighting the predictive value of LAS components, particularly PALS, in assessing the likelihood of HFpEF. The strong correlation between the HFA-PEFF score and LAS supports the use of PALS as a simple and sensitive ultrasound parameter for diagnosing HFpEF.

## Acknowledgement

The authors would like to thank the staff of the Cardiology Department and the Hematology and Biology Department at the Internal Security Forces Hospital of Marsa, as well as all the patients who participated in this study.

## Conflicts of interest:

None declared.
